# Supplementary material for: Representation and quantification of module activity from omics data with rROMA
Source: NPJ Syst Biol Appl. 2024 Jan 19;10:8. doi: 10.1038/s41540-024-00331-x (PMC10799004; doi:10.1038/s41540-024-00331-x)
Supplement: Supplementary file 1 — Supplementary Files [file 41540_2024_331_MOESM1_ESM.pdf]

Supplementary Figures

The tables below correspond to the analyses discussed in the subsection entitled “Review of existing methods” of the "Results" section. All these results can be reproduced using the codes publicly available in the following github repository: https://github.com/sysbio-curie/rRoma_comp.git

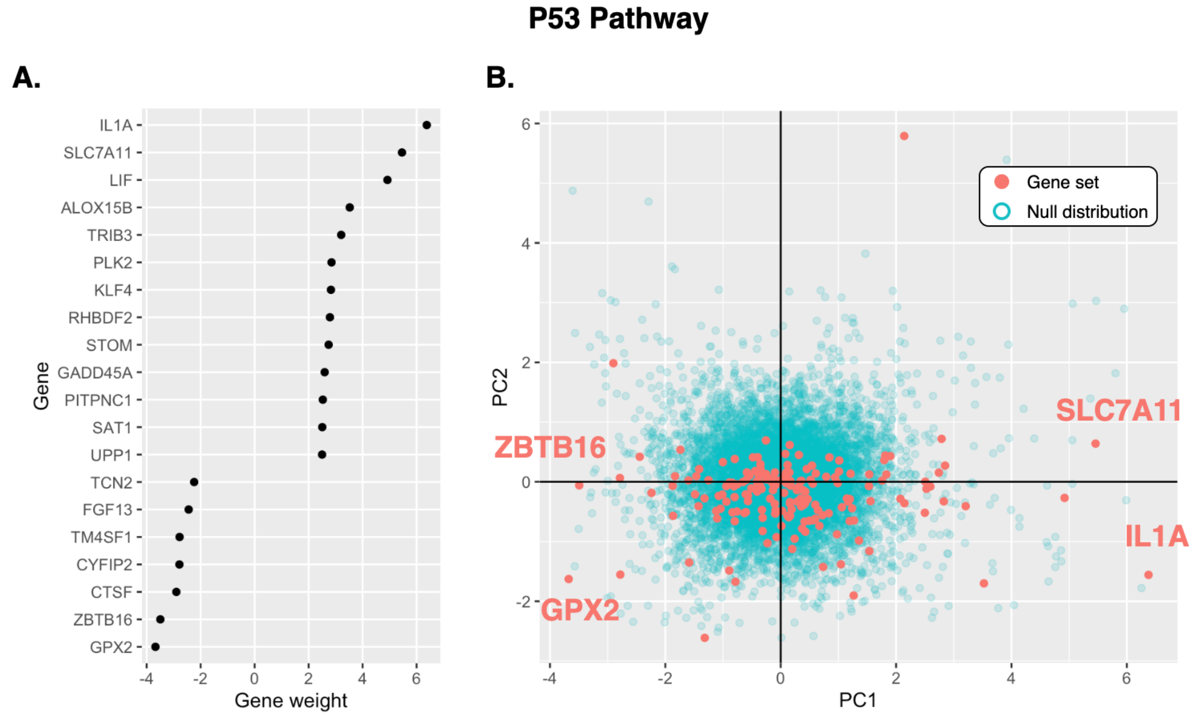

Supplementary Figure 1

Plots illustrating the contribution of genes to the *p53 Pathway* gene set activity score. The weights in panel A indicate the gene projections on PC1, limited to the genes that have the greatest contribution to the observed variation in the *p53 Pathway* gene set. In panel B the genes of the *p53 Pathway* gene set are represented in the PCA space. Red dots are genes from the gene set, blue dots show randomly selected genes used to generate a null distribution.

Supplementary Tables

Representation and quantification Of Module Activity from omics data with rROMA

The tables below correspond to the analyses discussed in the subsection entitled “Comparison with state-of-art methods” of the "Results" section. All these results can be reproduced using the codes publicly available in the following github repository: https://github.com/sysbio-curie/rRoma_comp.git

Supplementary Table 1. Statistical comparison of pathway activity scores calculated with PLAGE between CF and non-CF samples.
Student's t-Test Results for Pathway Activity Score Distributions. Pathways are considered significantly dysregulated between the two groups if the adjusted p-value for the statistical test is less than 0.05.

Pathway	Statistic	Adjusted P value	Significantly dysregulated
PI3K AKT MTOR SIGNALING	-10.46611192	8.99E-05	*
HYPOXIA	-7.8728174	0.00075734	*
UV RESPONSE DN	-7.393753306	0.001295239	*
REACTIVE OXYGEN SPECIES PATHWAY	7.07918733	0.00075734	*
COAGULATION	6.523385924	0.002725546	*
FATTY ACID METABOLISM	-6.401406048	0.00136566	*
ESTROGEN RESPONSE EARLY	6.198136791	0.00136566	*
GLYCOLYSIS	-6.172674124	0.001794162	*
TGF BETA SIGNALING	6.029963416	0.002209614	*
KRAS SIGNALING DN	6.020258131	0.002209614	*
ESTROGEN RESPONSE LATE	-5.985796397	0.002209614	*
IL2 STAT5 SIGNALING	-5.755082332	0.002209614	*
EPITHELIAL MESENCHYMAL TRANSITION	-5.551665307	0.003027189	*
P53 PATHWAY	-5.480280849	0.002209614	*
ANDROGEN RESPONSE	5.09293922	0.002781229	*
IL6 JAK STAT3 SIGNALING	-5.009265363	0.004247703	*
MYOGENESIS	-4.978020832	0.002209614	*
TNFA SIGNALING VIA NFKB	4.93284727	0.003240277	*
APOPTOSIS	-4.874667004	0.003161515	*
INTERFERON ALPHA RESPONSE	-4.71930024	0.004247703	*
ALLOGRAFT REJECTION	-4.627287252	0.003240277	*
MTORC1 SIGNALING	4.598470241	0.003730526	*
XENOBIOTIC METABOLISM	-4.381111694	0.004392226	*

HEME METABOLISM	4.353887063	0.003587087	*
COMPLEMENT	-4.271399831	0.004591059	*
INTERFERON GAMMA RESPONSE	4.266070731	0.005033633	*
PROTEIN SECRETION	4.257796239	0.004190017	*
KRAS SIGNALING UP	-4.174121101	0.004392226	*
NOTCH SIGNALING	-4.140378465	0.007597934	*
CHOLESTEROL HOMEOSTASIS	4.106650288	0.004247703	*
DNA REPAIR	-3.965790459	0.004591059	*
UV RESPONSE UP	-3.888100632	0.005277671	*
MYC TARGETS V1	-3.626084376	0.007269969	*
APICAL JUNCTION	-3.622244443	0.007597934	*
INFLAMMATORY RESPONSE	3.587373278	0.009333126	*
BILE ACID METABOLISM	-3.531025295	0.009421234	*
MITOTIC SPINDLE	3.105800306	0.015085794	*
UNFOLDED PROTEIN RESPONSE	3.015138925	0.017289806	*
PEROXISOME	-2.990445541	0.017956987	*
MYC TARGETS V2	-2.986092296	0.017956987	*
ADIPOGENESIS	2.943158211	0.018105408	*
SPERMATOGENESIS	-2.909355138	0.018630292	*
APICAL SURFACE	-2.719313819	0.025156513	*
PANCREAS BETA CELLS	-2.71544958	0.039886884	*
WNT BETA CATENIN SIGNALING	2.715328617	0.029414302	*
E2F TARGETS	1.999335047	0.084842798	
G2M CHECKPOINT	1.514602213	0.178499728	
HEDGEHOG SIGNALING	-1.452107094	0.184552367	
ANGIOGENESIS	0.512691922	0.634433744	
OXIDATIVE PHOSPHORYLATION	0.016424238	0.987238767	

Supplementary Table 2. Statistical comparison of pathway activity scores calculated with GSVA between CF and non-CF samples.

Student's t-Test Results for Pathway Activity Score Distributions. Pathways are considered significantly dysregulated between the two groups if the adjusted p-value for the statistical test is less than 0.05.

Pathway	Statistic	Adjusted P value	Significantly dysregulated
CHOLESTEROL HOMEOSTASIS	-4.2583141	0.07193158	
PANCREAS BETA CELLS	-3.9480733	0.07193158	
UV RESPONSE DN	-3.2188375	0.19919194	
NOTCH SIGNALING	-2.8809052	0.21576355	
MYC TARGETS V1	-2.3538376	0.44680405	
UNFOLDED PROTEIN RESPONSE	-2.1550241	0.44680405	
GLYCOLYSIS	-2.0066981	0.44680405	
PROTEIN SECRETION	-1.8927758	0.44680405	
HYPOXIA	-1.867643	0.44680405	
EPITHELIAL MESENCHYMAL TRANSITION	-1.7993547	0.44680405	
MYC TARGETS V2	-1.7300186	0.44680405	
SPERMATOGENESIS	1.72543495	0.44680405	
INTERFERON ALPHA RESPONSE	1.69433708	0.44680405	
ANDROGEN RESPONSE	-1.6491774	0.44680405	
UV RESPONSE UP	-1.6179378	0.44680405	
FATTY ACID METABOLISM	-1.5617	0.44680405	
ESTROGEN RESPONSE LATE	-1.5614345	0.44680405	
ADIPOGENESIS	-1.4662964	0.50851495	
INTERFERON GAMMA RESPONSE	1.3617774	0.54340283	
APICAL JUNCTION	-1.317758	0.54604351	
KRAS SIGNALING UP	-1.2858257	0.54604351	
G2M CHECKPOINT	-1.2371076	0.56983804	
MTORC1 SIGNALING	-1.1950031	0.56983804	
APOPTOSIS	-1.1605079	0.57620521	
OXIDATIVE PHOSPHORYLATION	-1.0679258	0.57869956	
WNT BETA CATENIN SIGNALING	-1.0671365	0.57869956	
KRAS SIGNALING DN	-1.0518503	0.57869956	
XENOBIOTIC METABOLISM	-1.0278476	0.57869956	
INFLAMMATORY RESPONSE	1.01173869	0.57869956	
P53 PATHWAY	-0.9558808	0.59549635	
APICAL SURFACE	-0.9403294	0.59549635	
PI3K AKT MTOR SIGNALING	-0.9018287	0.60688686	
COAGULATION	0.76761212	0.6984566	

HEDGEHOG SIGNALING	-0.7123355	0.70878901	
COMPLEMENT	0.70641358	0.70878901	
TNFA SIGNALING VIA NFKB	0.56220245	0.81521982	
IL6 JAK STAT3 SIGNALING	0.46463879	0.8812956	
ANGIOGENESIS	0.36811662	0.93619057	
ALLOGRAFT REJECTION	-0.3562653	0.93619057	
BILE ACID METABOLISM	0.30073265	0.94089833	
E2F TARGETS	-0.2956724	0.94089833	
DNA REPAIR	-0.2299608	0.94089833	
MYOGENESIS	-0.193361	0.94089833	
PEROXISOME	0.18899406	0.94089833	
REACTIVE OXYGEN SPECIES PATHWAY	-0.182344	0.94089833	
HEME METABOLISM	0.1736355	0.94089833	
ESTROGEN RESPONSE EARLY	0.12585917	0.94591243	
IL2 STAT5 SIGNALING	0.11885977	0.94591243	
TGF BETA SIGNALING	-0.0343818	0.98308987	
MITOTIC SPINDLE	0.02175825	0.98308987	

Supplementary Table 3. Statistical comparison of pathway activity scores calculated with ssGSEA between CF and non-CF samples.

Student's t-Test Results for Pathway Activity Score Distributions. Pathways are considered significantly dysregulated between the two groups if the adjusted p-value for the statistical test is less than 0.05.

Pathway	Statistic	Adjusted P value	Significantly dysregulated
NOTCH SIGNALING	-4.822276729	0.04275294	*
CHOLESTEROL HOMEOSTASIS	-3.939652225	0.07446598	
ANDROGEN RESPONSE	-3.027945159	0.28261421	
HEME METABOLISM	-2.874390793	0.27655661	
PANCREAS BETA CELLS	-2.633804043	0.32141782	
HYPOXIA	-2.605093214	0.29373994	
ADIPOGENESIS	-2.222870145	0.3596081	
UV RESPONSE DN	-2.101593997	0.3596081	
COMPLEMENT	2.094793106	0.3596081	
MTORC1 SIGNALING	-1.689282688	0.54369326	
ESTROGEN RESPONSE LATE	-1.560325773	0.54369326	
MITOTIC SPINDLE	-1.52001676	0.54369326	
INTERFERON ALPHA RESPONSE	1.494126497	0.54369326	
IL6 JAK STAT3 SIGNALING	1.493703475	0.54369326	
APICAL SURFACE	-1.48996321	0.54369326	
UNFOLDED PROTEIN RESPONSE	-1.448347407	0.54369326	
ALLOGRAFT REJECTION	1.436598765	0.54369326	
INTERFERON GAMMA RESPONSE	1.385613922	0.544968	
OXIDATIVE PHOSPHORYLATION	-1.347194957	0.54814073	
G2M CHECKPOINT	-1.299985575	0.56582101	
SPERMATOGENESIS	1.190281313	0.61087181	
ANGIOGENESIS	-1.154482263	0.61087181	
APICAL JUNCTION	-1.154400609	0.61087181	
BILE ACID METABOLISM	1.033383017	0.68799315	
EPITHELIAL MESENCHYMAL TRANSITION	-0.990347439	0.68799315	
PEROXISOME	-0.939384013	0.68799315	
IL2 STAT5 SIGNALING	0.937612401	0.68799315	
KRAS SIGNALING DN	-0.904521735	0.68970966	
GLYCOLYSIS	-0.881354011	0.68970966	
P53 PATHWAY	-0.851234529	0.70120444	
TNFA SIGNALING VIA NFKB	0.813738497	0.70478742	
FATTY ACID METABOLISM	-0.763759572	0.72564741	

MYC TARGETS V1	-0.69666021	0.75112783	
WNT BETA CATENIN SIGNALING	-0.641733838	0.75112783	
KRAS SIGNALING UP	-0.641198103	0.75112783	
DNA REPAIR	-0.638262137	0.75112783	
ESTROGEN RESPONSE EARLY	0.620928519	0.75112783	
MYC TARGETS V2	0.447493519	0.85189242	
COAGULATION	0.413602317	0.85189242	
PI3K AKT MTOR SIGNALING	-0.408323774	0.85189242	
INFLAMMATORY RESPONSE	0.384525556	0.85189242	
XENOBIOTIC METABOLISM	-0.3241693	0.85189242	
PROTEIN SECRETION	-0.319039849	0.85189242	
MYOGENESIS	-0.303886448	0.85189242	
E2F TARGETS	-0.288837994	0.85189242	
TGF BETA SIGNALING	-0.274424396	0.85189242	
APOPTOSIS	0.259404257	0.85189242	
HEDGEHOG SIGNALING	0.195327712	0.88483751	
UV RESPONSE UP	0.107348146	0.93534308	
REACTIVE OXYGEN SPECIES PATHWAY	-0.051128914	0.9602459	

Supplementary Table 4. Statistical comparison of pathway activity scores calculated with zscore between CF and non-CF samples.

Student's t-Test Results for Pathway Activity Score Distributions. Pathways are considered significantly dysregulated between the two groups if the adjusted p-value for the statistical test is less than 0.05.

Pathway	Statistic	Adjusted P value	Significantly dysregulated
COMPLEMENT	3.788848987	0.173669	
COAGULATION	3.304803741	0.173669	
IL6 JAK STAT3 SIGNALING	3.205733293	0.173669	
INTERFERON GAMMA RESPONSE	3.105616639	0.18057298	
INTERFERON ALPHA RESPONSE	2.952865385	0.18057298	
APOPTOSIS	2.890920654	0.18057298	
BILE ACID METABOLISM	2.614225266	0.18057298	
IL2 STAT5 SIGNALING	2.550598951	0.18057298	
XENOBIOTIC METABOLISM	2.244838507	0.26331355	
MYC TARGETS V2	2.164661646	0.26331355	
SPERMATOGENESIS	2.112265788	0.26331355	
REACTIVE OXYGEN SPECIES PATHWAY	2.044909387	0.26331355	
INFLAMMATORY RESPONSE	2.020138926	0.26331355	
UV RESPONSE UP	1.993919965	0.26331355	
TNFA SIGNALING VIA NFKB	1.961476576	0.26331355	
DNA REPAIR	1.898797185	0.27238808	
PANCREAS BETA CELLS	-1.847959672	0.29557998	
ALLOGRAFT REJECTION	1.761602037	0.30595006	
PI3K AKT MTOR SIGNALING	1.730401323	0.30595006	
ADIPOGENESIS	1.712179724	0.30595006	
ESTROGEN RESPONSE EARLY	1.603024678	0.32268495	
MYOGENESIS	1.601335471	0.32268495	
APICAL JUNCTION	1.593822751	0.32268495	
PEROXISOME	1.483172975	0.35176115	
FATTY ACID METABOLISM	1.344554835	0.42130856	
HEME METABOLISM	1.324562043	0.42130856	
P53 PATHWAY	1.286788584	0.42130856	
MTORC1 SIGNALING	1.26357283	0.42130856	
MITOTIC SPINDLE	1.174778007	0.45016383	
ANDROGEN RESPONSE	-1.151577109	0.45016383	
OXIDATIVE PHOSPHORYLATION	1.145524498	0.45016383	
GLYCOLYSIS	1.052927554	0.50680367	

TGF BETA SIGNALING	0.927949972	0.54645304	
UNFOLDED PROTEIN RESPONSE	0.892027261	0.54645304	
WNT BETA CATENIN SIGNALING	0.861681448	0.54645304	
HYPOXIA	0.86160632	0.54645304	
APICAL SURFACE	-0.818300149	0.54645304	
HEDGEHOG SIGNALING	0.816329222	0.54645304	
UV RESPONSE DN	0.809726604	0.54645304	
ESTROGEN RESPONSE LATE	0.791436852	0.54645304	
PROTEIN SECRETION	0.776510309	0.54645304	
KRAS SIGNALING UP	0.76673456	0.54645304	
MYC TARGETS V1	0.753222911	0.54645304	
CHOLESTEROL HOMEOSTASIS	0.528572443	0.68680887	
G2M CHECKPOINT	0.512939011	0.68680887	
E2F TARGETS	0.494311477	0.68680887	
EPITHELIAL MESENCHYMAL TRANSITION	0.288761761	0.83056981	
NOTCH SIGNALING	-0.176294088	0.88227279	
ANGIOGENESIS	0.175381987	0.88227279	
KRAS SIGNALING DN	0.121288866	0.90594959	

Supplementary Table 5. Statistical comparison of pathway activity scores calculated with rROMA between CF and non-CF samples.

Student's t-Test Results for Pathway Activity Score Distributions. Pathways are considered significantly dysregulated between the two groups if the adjusted p-value for the statistical test is less than 0.05.

Pathway	Statistic	Adjusted P value	Significantly dysregulated
ESTROGEN RESPONSE LATE	7.131437216	0.001952235	*
MYOGENESIS	-5.601443178	0.008141925	*
HEME METABOLISM	4.736443887	0.028809085	*
ESTROGEN RESPONSE EARLY	4.413501708	0.028809085	*
COAGULATION	-3.816855131	0.037587521	*
UV RESPONSE UP	-3.730322919	0.037587521	*
MITOTIC SPINDLE	3.554284122	0.038030903	*
XENOBIOTIC METABOLISM	-3.466997983	0.038030903	*
UV RESPONSE DN	3.342873947	0.055838971	
INFLAMMATORY RESPONSE	3.232577168	0.055838971	
APOPTOSIS	-3.152959554	0.061828826	
FATTY ACID METABOLISM	3.044322009	0.061828826	
MTORC1 SIGNALING	2.939218257	0.061828826	
DNA REPAIR	-2.738587253	0.087082176	
G2M CHECKPOINT	-2.669605161	0.113184688	
MYC TARGETS V1	-2.594553432	0.091871061	
TNFA SIGNALING VIA NFKB	2.465165249	0.104317251	
APICAL SURFACE	-2.375497717	0.120686036	
IL6 JAK STAT3 SIGNALING	-2.328231601	0.129225792	
BILE ACID METABOLISM	-2.257768415	0.138644473	
PANCREAS BETA CELLS	-2.248955508	0.151024286	
COMPLEMENT	2.059780211	0.151891938	
PI3K AKT MTOR SIGNALING	-2.010708805	0.186229154	
KRAS SIGNALING UP	1.882074317	0.186229154	
TGF BETA SIGNALING	1.629648386	0.29244456	
ALLOGRAFT REJECTION	-1.629529591	0.27332323	
ADIPOGENESIS	1.615358044	0.29244456	
IL2 STAT5 SIGNALING	-1.377609348	0.361172315	
GLYCOLYSIS	1.255902794	0.409835329	
INTERFERON GAMMA RESPONSE	-1.230145965	0.41162513	
KRAS SIGNALING DN	-1.054925058	0.515225312	
PEROXISOME	1.045174781	0.515225312	

OXIDATIVE PHOSPHORYLATION	-0.970035855	0.542503255	
NOTCH SIGNALING	0.913835482	0.555316441	
PROTEIN SECRETION	-0.902687054	0.555316441	
ANGIOGENESIS	-0.839287169	0.571260954	
APICAL JUNCTION	0.810192348	0.571260954	
ANDROGEN RESPONSE	0.798272977	0.571260954	
REACTIVE OXIGEN SPECIES PATHWAY	0.798071856	0.571260954	
E2F TARGETS	0.700088474	0.620234564	
MYC TARGETS V2	-0.6860327	0.620234564	
WNT BETA CATENIN SIGNALING	-0.576428498	0.687031565	
P53 PATHWAY	-0.550901101	0.692630774	
SPERMATOGENESIS	0.527650003	0.693614074	
HYPOXIA	0.287591522	0.830525007	
CHOLESTEROL HOMEOSTASIS	-0.27897424	0.830525007	
UNFOLDED PROTEIN RESPONSE	0.271875876	0.830525007	
EPITHELIAL MESENCHYMAL TRANSITION	-0.265134318	0.830525007	
HEDGEHOG SIGNALING	-0.213098959	0.853969693	
INTERFERON ALPHA RESPONSE	0.184922983	0.85705999	

Supplementary Table 6. Statistical comparison of pathway activity scores calculated with PLAGE of six healthy control samples before and after treatment with IL17+ TNFα. Student's t-Test Results for Pathway Activity Score Distributions. Pathways are considered significantly dysregulated between the two groups if the adjusted p-value for the statistical test is less than 0.05.

Pathway	Statistic	Adjusted P value	Significantly dysregulated
MTORC1 SIGNALING	23.97628217	6.57E-07	*
ANDROGEN RESPONSE	-21.18949886	6.35E-07	*
UNFOLDED PROTEIN RESPONSE	19.67751982	1.52E-07	*
REACTIVE OXYGEN SPECIES PATHWAY	-16.16542695	6.57E-07	*
HEME METABOLISM	-15.99100779	1.20E-06	*
COMPLEMENT	-15.32482203	1.46E-06	*
UV RESPONSE UP	-14.6742543	6.57E-07	*
INTERFERON GAMMA RESPONSE	-14.51296258	2.05E-06	*
APOPTOSIS	13.87739804	1.20E-06	*
MYOGENESIS	11.59574122	2.05E-06	*
KRAS SIGNALING DN	-11.56843781	3.12E-06	*
P53 PATHWAY	11.11281476	3.12E-06	*
IL2 STAT5 SIGNALING	10.8442579	3.12E-06	*
ALLOGRAFT REJECTION	10.7495014	3.64E-06	*
INFLAMMATORY RESPONSE	10.59558797	6.58E-06	*
FATTY ACID METABOLISM	-10.45578795	4.44E-06	*
KRAS SIGNALING UP	10.42435963	3.64E-06	*
**TNFA SIGNALING VIA NFKB**	**10.14226248**	**2.12E-05**	*
INTERFERON ALPHA RESPONSE	-9.630064861	1.37E-05	*
HYPOXIA	9.234160242	3.26E-05	*
ESTROGEN RESPONSE LATE	9.230380159	2.09E-05	*
ESTROGEN RESPONSE EARLY	9.223821357	1.37E-05	*
IL6 JAK STAT3 SIGNALING	9.080910218	3.42E-05	*
COAGULATION	8.214494819	2.12E-05	*
CHOLESTEROL HOMEOSTASIS	8.112680921	3.15E-05	*
APICAL SURFACE	7.99178274	0.00028709	*
GLYCOLYSIS	7.909647544	0.000273781	*
XENOBIOTIC METABOLISM	7.258733294	5.62E-05	*
ANGIOGENESIS	7.067732811	0.000215055	*
WNT BETA CATENIN SIGNALING	6.622902795	0.000163963	*
TGF BETA SIGNALING	6.578875289	0.000150346	*
HEDGEHOG SIGNALING	-6.544141701	0.000138607	*

UV RESPONSE DN	-5.730277907	0.00028709	*
BILE ACID METABOLISM	-5.712056227	0.00028709	*
EPITHELIAL MESENCHYMAL TRANSITION	5.054325583	0.002064033	*
MYC TARGETS V1	4.897693206	0.001441583	*
APICAL JUNCTION	-4.623149965	0.005585846	*
ADIPOGENESIS	-4.446958077	0.004524336	*
PI3K AKT MTOR SIGNALING	-4.250015561	0.008384915	*
PROTEIN SECRETION	4.122533265	0.004524336	*
G2M CHECKPOINT	3.917378257	0.006445862	*
PEROXISOME	-3.853005467	0.005504056	*
MYC TARGETS V2	3.850342435	0.005804163	*
SPERMATOGENESIS	3.021432592	0.014656873	*
E2F TARGETS	2.739812933	0.02482046	*
PANCREAS BETA CELLS	1.941064239	0.088889285	
NOTCH SIGNALING	1.267391488	0.251693937	
DNA REPAIR	-0.476866549	0.674323923	
MITOTIC SPINDLE	0.436684947	0.687714576	
OXIDATIVE PHOSPHORYLATION	0.103739457	0.919822292	

Supplementary Table 7. Statistical comparison of pathway activity scores calculated with GSVA of six healthy control samples before and after treatment with IL17+ TNFα. Student's t-Test Results for Pathway Activity Score Distributions. Pathways are considered significantly dysregulated between the two groups if the adjusted p-value for the statistical test is less than 0.05.

Pathway	Statistic	Adjusted P value	Significantly dysregulated
ALLOGRAFT REJECTION	-11.528991	3.56E-05	*
MTORC1 SIGNALING	-10.796956	0.00030432	*
UNFOLDED PROTEIN RESPONSE	-10.024098	0.0001938	*
KRAS SIGNALING DN	8.22868012	0.00024089	*
**TNFA SIGNALING VIA NFKB**	**-7.7666112**	**0.00046628**	*****
INFLAMMATORY RESPONSE	-7.4756359	0.00030432	*
BILE ACID METABOLISM	7.0978739	0.00071726	*
COMPLEMENT	-7.0526869	0.00202716	*
PI3K AKT MTOR SIGNALING	-6.8613494	0.00071726	*
APOPTOSIS	-6.6862449	0.00301596	*
INTERFERON GAMMA RESPONSE	-6.44951	0.00301596	*
UV RESPONSE UP	-5.8253782	0.00232236	*
MYC TARGETS V2	-5.2595049	0.00601904	*
IL2 STAT5 SIGNALING	-4.9906616	0.00301596	*
INTERFERON ALPHA RESPONSE	-4.845393	0.00735427	*
IL6 JAK STAT3 SIGNALING	-4.7752027	0.00360094	*
GLYCOLYSIS	-4.6779849	0.00436264	*
HYPOXIA	-4.6004439	0.00358404	*
KRAS SIGNALING UP	-4.3528744	0.00524539	*
TGF BETA SIGNALING	-4.2853398	0.00530355	*
EPITHELIAL MESENCHYMAL TRANSITION	-4.1567049	0.00758189	*
CHOLESTEROL HOMEOSTASIS	-4.0149289	0.00700985	*
MYC TARGETS V1	-3.2807504	0.02220357	*
REACTIVE OXYGEN SPECIES PATHWAY	-2.8239818	0.04405512	*
P53 PATHWAY	-2.669379	0.06844252	
XENOBIOTIC METABOLISM	-2.4051107	0.07999824	
SPERMATOGENESIS	2.3562526	0.07887432	
PEROXISOME	2.2514538	0.09035527	
E2F TARGETS	-2.1952378	0.09115244	
DNA REPAIR	-2.0936935	0.10463397	
G2M CHECKPOINT	-2.0278261	0.11488578	
COAGULATION	-1.9018244	0.14165247	

PANCREAS BETA CELLS	1.70470049	0.18376602	
ANDROGEN RESPONSE	-1.6566487	0.1975407	
APICAL JUNCTION	-1.5758818	0.2318984	
ESTROGEN RESPONSE LATE	-1.5023118	0.2318984	
HEME METABOLISM	1.43256202	0.25358718	
OXIDATIVE PHOSPHORYLATION	-1.3944122	0.2678511	
FATTY ACID METABOLISM	1.35329572	0.27499478	
APICAL SURFACE	-1.1712432	0.34824937	
ADIPOGENESIS	1.06191354	0.38225943	
ANGIOGENESIS	-1.0434196	0.38514058	
PROTEIN SECRETION	-0.9486378	0.42516882	
NOTCH SIGNALING	0.90391186	0.44012109	
MYOGENESIS	0.82907339	0.48383025	
HEDGEHOG SIGNALING	0.56086451	0.63296855	
UV RESPONSE DN	0.54989462	0.63296855	
MITOTIC SPINDLE	-0.1524362	0.92000734	
WNT BETA CATENIN SIGNALING	0.0835491	0.95434108	
ESTROGEN RESPONSE EARLY	-0.0376907	0.97077707	

Supplementary Table 8. Statistical comparison of pathway activity scores calculated with ssGSEA of six healthy control samples before and after treatment with IL17+ TNFα. Student's t-Test Results for Pathway Activity Score Distributions. Pathways are considered significantly dysregulated between the two groups if the adjusted p-value for the statistical test is less than 0.05.

Pathway	Statistic	Adjusted P value	Significantly dysregulated
MTORC1 SIGNALING	-14.8652	2.15E-06	*
ALLOGRAFT REJECTION	-9.8895625	5.16E-05	*
INFLAMMATORY RESPONSE	-9.4466069	5.16E-05	*
UNFOLDED PROTEIN RESPONSE	-8.8623377	0.00030037	*
**TNFA SIGNALING VIA NFKB**	**-8.6467645**	**0.00011558**	*****
INTERFERON GAMMA RESPONSE	-8.5816246	0.00020606	*
COMPLEMENT	-8.2639143	0.00030037	*
UV RESPONSE UP	-7.8951432	0.00014496	*
KRAS SIGNALING UP	-7.3285334	0.00020606	*
KRAS SIGNALING DN	7.04847381	0.00024094	*
PEROXISOME	6.97739747	0.00053309	*
IL2 STAT5 SIGNALING	-6.896164	0.00026078	*
IL6 JAK STAT3 SIGNALING	-6.3378707	0.00041921	*
INTERFERON ALPHA RESPONSE	-6.2746741	0.00092926	*
BILE ACID METABOLISM	5.66369733	0.00363838	*
ANGIOGENESIS	-4.854282	0.00275195	*
GLYCOLYSIS	-4.8451149	0.00363838	*
CHOLESTEROL HOMEOSTASIS	-4.6587943	0.00337786	*
HYPOXIA	-4.6015375	0.00721322	*
MYC TARGETS V2	-4.4137412	0.00487762	*
APOPTOSIS	-3.9749204	0.01286446	*
EPITHELIAL MESENCHYMAL TRANSITION	-3.7143598	0.01803763	*
MYC TARGETS V1	-3.6561644	0.01057218	*
TGF BETA SIGNALING	-3.2646516	0.02286195	*
PI3K AKT MTOR SIGNALING	-3.2265993	0.03162186	*
XENOBIOTIC METABOLISM	-2.8379	0.03435124	*
COAGULATION	-2.7105764	0.0405898	*
ADIPOGENESIS	2.61844552	0.05358023	
HEME METABOLISM	2.34868274	0.08654952	
UV RESPONSE DN	2.22855019	0.08654952	
NOTCH SIGNALING	1.80306974	0.16498129	
G2M CHECKPOINT	-1.8001229	0.16498129	

PANCREAS BETA CELLS	1.71697739	0.17753607	
APICAL SURFACE	-1.7111519	0.18013555	
APICAL JUNCTION	-1.5983901	0.22538696	
E2F TARGETS	-1.5384674	0.22189541	
P53 PATHWAY	-1.4996939	0.23802635	
DNA REPAIR	-1.4111277	0.25348116	
HEDGEHOG SIGNALING	1.28199806	0.29677197	
FATTY ACID METABOLISM	1.26140802	0.29950653	
PROTEIN SECRETION	1.16977066	0.33042359	
SPERMATOGENESIS	1.00449148	0.39773876	
REACTIVE OXYGEN SPECIES PATHWAY	-1.0020734	0.39773876	
OXIDATIVE PHOSPHORYLATION	0.89363488	0.44675842	
ESTROGEN RESPONSE LATE	-0.8872184	0.44773648	
ANDROGEN RESPONSE	-0.6629781	0.56800376	
WNT BETA CATENIN SIGNALING	0.48277189	0.68281661	
MYOGENESIS	0.2906963	0.8135398	
ESTROGEN RESPONSE EARLY	-0.266021	0.81416183	
MITOTIC SPINDLE	0.21288903	0.83845235	

Supplementary Table 9. Statistical comparison of pathway activity scores calculated with the zscore method of six healthy control samples before and after treatment with IL17+ TNFα.

Student's t-Test Results for Pathway Activity Score Distributions. Pathways are considered significantly dysregulated between the two groups if the adjusted p-value for the statistical test is less than 0.05.

Pathway	Statistic	Adjusted P value	Significantly dysregulated
INTERFERON GAMMA RESPONSE	-9.4940025	0.0001478	*
COMPLEMENT	-9.2705998	0.00014824	*
ALLOGRAFT REJECTION	-8.8820014	0.00031059	*
INFLAMMATORY RESPONSE	-8.1949826	0.00017486	*
IL6 JAK STAT3 SIGNALING	-7.3298999	0.00045592	*
MTORC1 SIGNALING	-7.116113	0.00033797	*
**TNFA SIGNALING VIA NFKB**	**-6.9872271**	**0.00059199**	*****
UNFOLDED PROTEIN RESPONSE	-6.7373839	0.00123356	*
INTERFERON ALPHA RESPONSE	-6.3299298	0.00059199	*
KRAS SIGNALING DN	5.16711344	0.00228779	*
KRAS SIGNALING UP	-4.5144759	0.0143162	*
CHOLESTEROL HOMEOSTASIS	-3.8964972	0.01588857	*
BILE ACID METABOLISM	3.76762084	0.01551586	*
IL2 STAT5 SIGNALING	-3.4905364	0.02088466	*
APOPTOSIS	-3.1980372	0.03381352	*
HYPOXIA	-3.1035209	0.0467283	*
MYC TARGETS V2	-2.9066222	0.0467283	*
EPITHELIAL MESENCHYMAL TRANSITION	-2.7588543	0.0841187	
GLYCOLYSIS	-2.6882932	0.07630969	
UV RESPONSE UP	-2.1979366	0.12833812	
MYC TARGETS V1	-2.1841344	0.12833812	
PI3K AKT MTOR SIGNALING	-2.1015977	0.15495029	
ANDROGEN RESPONSE	-1.9888106	0.16489903	
NOTCH SIGNALING	1.9312765	0.17791299	
TGF BETA SIGNALING	-1.7445133	0.22515484	
UV RESPONSE DN	1.5892387	0.28420775	
PANCREAS BETA CELLS	1.57044072	0.28420775	
PEROXISOME	1.51996224	0.28420775	
FATTY ACID METABOLISM	1.51764142	0.28420775	
HEDGEHOG SIGNALING	1.38398884	0.32335849	
REACTIVE OXYGEN SPECIES PATHWAY	-1.378934	0.32335849	

SPERMATOGENESIS	1.3384199	0.3290113	
MYOGENESIS	1.14471482	0.42436533	
PROTEIN SECRETION	-1.0993512	0.43739531	
HEME METABOLISM	1.02103719	0.48094415	
APICAL JUNCTION	-0.9705057	0.50045297	
E2F TARGETS	-0.858132	0.54239376	
MITOTIC SPINDLE	0.85576854	0.54239376	
G2M CHECKPOINT	-0.8178886	0.55487726	
ANGIOGENESIS	-0.7353067	0.59685757	
ESTROGEN RESPONSE EARLY	0.71856634	0.59685757	
ADIPOGENESIS	0.5266811	0.72626586	
COAGULATION	-0.4585249	0.75725393	
DNA REPAIR	-0.4453432	0.75725393	
ESTROGEN RESPONSE LATE	0.32065699	0.83928702	
WNT BETA CATENIN SIGNALING	0.15024036	0.95142924	
P53 PATHWAY	-0.136782	0.95142924	
APICAL SURFACE	-0.0880666	0.96969087	
XENOBIOTIC METABOLISM	0.06391567	0.96969087	
OXIDATIVE PHOSPHORYLATION	0.0080938	0.99370482	

Supplementary Table 10. The module matrix, output of ROMA applied to the dataset of six healthy control samples before and after treatment with IL17+ TNFα. L_1 and Median Exp values and their corresponding q-values are detailed for each pathway
Pathways with a ppv Median Exp lower than 0.05 were deemed as shifted, while those with a ppv L1 lower than 0.05 were overdispersed.

Pathway	Median Exp	ppv Median Exp	Significantly shifted	L1	ppv L1	Significantly overdispersed
**TNFA SIGNALING VIA NFKB**	**-1.152504513**	**0**	*	**0.61305497**	**0**	
IL6 JAK STAT3 SIGNALING	0.680197762	0	*	0.57757555	0	
INFLAMMATORY RESPONSE	-0.641300514	0	*	0.60987522	0	
EPITHELIAL MESENCHYMAL TRANSITION	0.635674014	0	*	0.37253042	0.02	
INTERFERON GAMMA RESPONSE	0.554792055	0	*	0.320295	0.11	
INTERFERON ALPHA RESPONSE	0.533633402	0	*	0.45407218	0	
CHOLESTEROL HOMEOSTASIS	0.462009532	0	*	0.28227273	0.82	
MTORC1 SIGNALING	-0.408573197	0	*	0.42159291	0	
E2F TARGETS	0.36190834	0	*	0.22002875	0.98	
BILE ACID METABOLISM	-0.347768789	0	*	0.33412996	0.3	
MYC TARGETS V2	0.341086164	0	*	0.32016549	0.66	
TGF BETA SIGNALING	0.336414092	0	*	0.27679725	0.85	
G2M CHECKPOINT	-0.330321817	0	*	0.21013666	0.99	
HYPOXIA	0.324540085	0	*	0.3041	0.17	
MYC TARGETS V1	0.319570237	0	*	0.41528389	0	
UNFOLDED PROTEIN RESPONSE	-0.314415091	0	*	0.37582862	0.09	
COMPLEMENT	-0.262171341	0	*	0.43897704	0	
ALLOGRAFT REJECTION	-0.221927173	0	*	0.40965289	0	
PI3K AKT MTOR SIGNALING	-0.220918753	0	*	0.37134795	0.11	
APICAL JUNCTION	-0.207837474	0	*	0.33469311	0.05	
APOPTOSIS	-0.200246604	0	*	0.38205614	0.04	
GLYCOLYSIS	-0.193104771	0	*	0.29406968	0.3	
PANCREAS BETA CELLS	0.186458679	0.02	*	0.38590162	0.43	
SPERMATOGENESIS	0.161337599	0	*	0.24686815	0.8	
IL2 STAT5 SIGNALING	0.160215922	0	*	0.43651463	0	
DNA REPAIR	-0.158446262	0	*	0.30534189	0.28	
UV RESPONSE DN	0.152979703	0	*	0.32894881	0.16	
UV RESPONSE UP	-0.143156243	0.02	*	0.51848739	0.01	*
NOTCH SIGNALING	0.131557173	0.12		0.36726051	0.6	
COAGULATION	-0.118514596	0.05		0.35770803	0.09	
PROTEIN SECRETION	-0.116612281	0.1		0.51586828	0	*
P53 PATHWAY	0.107614967	0.06		0.46124862	0	*

KRAS SIGNALING UP	0.104266008	0.05		0.48484275	0	*
PEROXISOME	-0.096360714	0.11		0.40054009	0.05	
APICAL SURFACE	0.096140612	0.26		0.43962115	0.25	
MITOTIC SPINDLE	-0.092916391	0.1		0.22401655	0.96	
XENOBIOTIC METABOLISM	0.091540856	0.1		0.4112217	0	*
HEME METABOLISM	-0.076539169	0.27		0.34653474	0.06	
REACTIVE OXIGEN SPECIES PATHWAY	0.076165766	0.42		0.43463643	0.23	
ANDROGEN RESPONSE	0.069540537	0.45		0.43002442	0.02	
ADIPOGENESIS	0.066342693	0.38		0.3697384	0.03	
HEDGEHOG SIGNALING	-0.057635097	0.67		0.36471211	0.56	
FATTY ACID METABOLISM	0.049960259	0.51		0.43713118	0.02	
KRAS SIGNALING DN	0.04489938	0.61		0.35638899	0.04	
OXIDATIVE PHOSPHORYLATION	-0.043240116	0.62		0.29224508	0.32	
MYOGENESIS	-0.040757927	0.63		0.25880506	0.73	
WNT BETA CATENIN SIGNALING	-0.035258794	0.72		0.37002235	0.51	
ANGIOGENESIS	-0.028531281	0.8		0.44517629	0.34	
ESTROGEN RESPONSE LATE	0.027287362	0.78		0.48470835	0	*
ESTROGEN RESPONSE EARLY	-0.012195462	0.89		0.40678387	0	*
