# Supplementary material for: Representation and quantification of module activity from omics data with rROMA
Source: NPJ Syst Biol Appl. 2024 Jan 19;10:8. doi: 10.1038/s41540-024-00331-x (PMC10799004; doi:10.1038/s41540-024-00331-x)
Supplement: Supplementary file 2 — Reporting summary [file 41540_2024_331_MOESM2_ESM.pdf]

nature portfolio

Corresponding author(s): MARTIGNETTI Loredana

Last updated by author(s): 30th October 2023

Reporting Summary

Nature Portfolio wishes to improve the reproducibility of the work that we publish. This form provides structure for consistency and transparency in reporting. For further information on Nature Portfolio policies, see our Editorial Policies and the Editorial Policy Checklist.

Statistics

For all statistical analyses, confirm that the following items are present in the figure legend, table legend, main text, or Methods section.

n/a	Confirmed	
☐	☒	The exact sample size (*n*) for each experimental group/condition, given as a discrete number and unit of measurement
☐	☒	A statement on whether measurements were taken from distinct samples or whether the same sample was measured repeatedly
☐	☒	The statistical test(s) used AND whether they are one- or two-sided<br>*Only common tests should be described solely by name; describe more complex techniques in the Methods section.*
☐	☒	A description of all covariates tested
☐	☒	A description of any assumptions or corrections, such as tests of normality and adjustment for multiple comparisons
☐	☒	A full description of the statistical parameters including central tendency (e.g. means) or other basic estimates (e.g. regression coefficient) AND variation (e.g. standard deviation) or associated estimates of uncertainty (e.g. confidence intervals)
☐	☒	For null hypothesis testing, the test statistic (e.g. *F*, *t*, *r*) with confidence intervals, effect sizes, degrees of freedom and *P* value noted<br>*Give P values as exact values whenever suitable.*
☒	☐	For Bayesian analysis, information on the choice of priors and Markov chain Monte Carlo settings
☐	☒	For hierarchical and complex designs, identification of the appropriate level for tests and full reporting of outcomes
☒	☐	Estimates of effect sizes (e.g. Cohen's *d*, Pearson's *r*), indicating how they were calculated

Our web collection on statistics for biologists contains articles on many of the points above.

Policy information about availability of computer code

Data collection	The data used in this study has been downloaded from the NCBI GEO database (GEO accession ID GSE176121).
Data analysis	The computational analyses were performed in R 4.2.2 and all the code is available under a GNU General Public License V3 in a GitHub repository at the following url: https://github.com/sysbio-curie/rROMA. All results can be reproduced using the codes publicly available in the following github repository: https://github.com/sysbio-curie/rRoma_comp.git

For manuscripts utilizing custom algorithms or software that are central to the research but not yet described in published literature, software must be made available to editors and reviewers. We strongly encourage code deposition in a community repository (e.g. GitHub). See the Nature Portfolio guidelines for submitting code & software for further information.

Policy information about availability of data

All manuscripts must include a data availability statement. This statement should provide the following information, where applicable:

- Accession codes, unique identifiers, or web links for publicly available datasets
- A description of any restrictions on data availability
- For clinical datasets or third party data, please ensure that the statement adheres to our

The data utilized in this study has been downloaded from the NCBI GEO database (GEO accession ID GSE176121).

Policy information about studies with . See also policy information about and

Reporting on sex and gender	Baseline clinical characteristics of study subjects, including sex, was previously reported in ref. [27], (GEO accession ID GSE176121).
Reporting on race, ethnicity, or other socially relevant groupings	Race and ethnicity information has not e collected.
Population characteristics	Baseline clinical characteristics of study subjects, including age, was previously reported in ref. [27], (GEO accession ID GSE176121).
Recruitment	All participants (and their guardians, as applicable) provided written informed consent.
Ethics oversight	The studies were approved by the University of Iowa Institutional Review Board and the Research Ethics Committee of St. Vincent's University Hospital.

Note that full information on the approval of the study protocol must also be provided in the manuscript.

Please select the one below that is the best fit for your research. If you are not sure, read the appropriate sections before making your selection.

☒ Life sciences ☐ Behavioural & social sciences ☐ Ecological, evolutionary & environmental sciences

For a reference copy of the document with all sections, see nature.com/documents/nr-reporting-summary-flat.pdf

All studies must disclose on these points even when the disclosure is negative.

Sample size	we compared the transcriptomes of primary cultures of airway epithelial cells from patients (N=6) with those of healthy controls (N=6).
Data exclusions	No data were excluded from the analysis.
Replication	All results can be reproduced using the codes publicly available in the following github repository: https://github.com/sysbio-curie/rRoma_comp.git
Randomization	Adult participants with CF who had at least 1 G551D or R117H allele were enrolled.
Blinding	Experimental design was not blind and included labeled CF and control samples.

We require information from authors about some types of materials, experimental systems and methods used in many studies. Here, indicate whether each material, system or method listed is relevant to your study. If you are not sure if a list item applies to your research, read the appropriate section before selecting a response.

Materials & experimental systems

n/a	Involved in the study
☒	☐ Antibodies
☒	☐ Eukaryotic cell lines
☒	☐ Palaeontology and archaeology
☒	☐ Animals and other organisms
☐	☒ Clinical data
☒	☐ Dual use research of concern
☒	☐ Plants

Methods

n/a	Involved in the study
☒	☐ ChIP-seq
☒	☐ Flow cytometry
☒	☐ MRI-based neuroimaging

Policy information about clinical studies

All manuscripts should comply with the ICMJE guidelines for publication of clinical research and a completed CONSORT checklist must be included with all submissions.

Clinical trial registration	No clinical trial registration was cited in the original publication.
Study protocol	RNA was extracted using Qiagen RNeasy Lipid Tissue Mini Kit # 74804.<br>mRNA libraries were prepared using Illumina TruSeq stranded mRNA sample preparation kit (Illumina #RS-122-2101)
Data collection	From April 2012 until June 2016, in Iowa, all participants were enrolled.
Outcomes	NA

Seed stocks

Novel plant genotypes

Authentication
